# The genetic variation in the R1a clade among the Ashkenazi Levites’ Y chromosome

**DOI:** 10.1038/s41598-017-14761-7

**Published:** 2017-11-02

**Authors:** Doron M. Behar, Lauri Saag, Monika Karmin, Meir G. Gover, Jeffrey D. Wexler, Luisa Fernanda Sanchez, Elliott Greenspan, Alena Kushniarevich, Oleg Davydenko, Hovhannes Sahakyan, Levon Yepiskoposyan, Alessio Boattini, Stefania Sarno, Luca Pagani, Shai Carmi, Shay Tzur, Ene Metspalu, Concetta Bormans, Karl Skorecki, Mait Metspalu, Siiri Rootsi, Richard Villems

**Affiliations:** 1 Estonian Biocentre, Tartu, 51010 Estonia; 2 Genomic Research Center, Gene by Gene, Houston, 77008 Texas USA; 3 Independent Genetic Genealogy Researcher, Savyon, 5690500 Israel; 4 levitedna.org, Los Angeles, 90045 California, USA; 5 Institute of Genetics and Cytology, National Academy of Sciences of Belarus, 220072 Minsk, Belarus; 6 Laboratory of Ethnogenomics, Institute of Molecular Biology of National Academy of Sciences, Yerevan, 0014 Armenia; 7 Department of Biological, Geological and Environmental Sciences, University of Bologna, Bologna, 40126 Italy; 8 APE Lab, Dept. of Biology, University of Padova, 35121 Padova, Italy; 9 Braun School of Public Health and Community Medicine, The Hebrew University of Jerusalem, Jerusalem, 9112102 Israel; 10 Rambam Health Care Campus, Haifa, 3109601 Israel; 11 Department of Evolutionary Biology, Institute of Molecular and Cell Biology University of Tartu, Tartu, 51010 Estonia; 12 Ruth and Bruce Rappaport Faculty of Medicine, Technion-Israel Institute of Technology, Haifa, 3109601 Israel

## Abstract

Approximately 300,000 men around the globe self-identify as Ashkenazi Levites, of whom two thirds were previously shown to descend from a single male. The paucity of whole Y-chromosome sequences precluded conclusive identification of this ancestor’s age, geographic origin and migration patterns. Here, we report the variation of 486 Y-chromosomes within the Ashkenazi and non-Ashkenazi Levite R1a clade, other Ashkenazi Jewish paternal lineages, as well as non-Levite Jewish and non-Jewish R1a samples. Cumulatively, the emerging profile is of a Middle Eastern ancestor, self-affiliating as Levite, and carrying the highly resolved R1a-Y2619 lineage, which was likely a minor haplogroup among the Hebrews. A star-like phylogeny, coalescing similarly to other Ashkenazi paternal lineages, ~1,743 ybp, suggests it to be one of the Ashkenazi paternal founders; to have expanded as part of the overall Ashkenazi demographic expansion, without special relation to the Levite affiliation; and to have subsequently spread to non-Ashkenazi Levites.

## Introduction

Sometimes, it is worthwhile to go back to the beginning. The evolution of surnames, family names or last names varies around the world^1^. As human population increased, the use of surnames turned from a convenience, to a necessity, to a full set of customs and laws with each constituency or culture having its own rules as to how these names are formed, transmitted and used. One common practice held by many West Eurasian societies is that, upon marriage, the couple and their offspring would adopt the father’s surname after the given name. The fact that such a patrilineal surname system is deeply rooted in human culture is self-evident as patronyms (Son of Steven), serving as the first surnames, transformed throughout the generations into modern-day patronymic surnames (Stevenson). Geneticists have been quick to understand that the patrilineal surname system matches the inheritance mode of the Y chromosome^2–4^. Since the Y chromosome is transmitted only from fathers to their sons, it is an ideal genetic locus for purposes such as tracing paternal ancestry and genealogical relatedness among contemporary males^5^. The absence of Y chromosome recombination enables merging of all contemporary Y chromosome sequences into a single, ever-evolving, phylogenetic tree whose branches are often referred to as haplogroups. Recently, the availability of high-coverage Y chromosome sequences marked a significant transition in the field of phylogenetics, allowing the resolution and understanding of paternal demographic events that were heretofore obscure^4,6–10^.

Jewish surnames have not been different in their evolution and, among others, are currently comprised of Aramaic and Hebrew patronymic surnames, surnames representing a Diaspora residency or occupation, and modern day Hebraized names^11,12^. World Jewry, estimated at 14 million individuals, can be roughly divided into Ashkenazi and non-Ashkenazi Jews. The former group is considered to have been formed approximately 2,000 ybp and to account for approximately 75% of contemporary Jews^13^. Notably, despite this remote split, Ashkenazi and non-Ashkenazi communities share two designations representing the two Jewish priesthood lineages, Levite (Levi, in Hebrew) and Cohen, whose etymologies relate to the Biblical male ancestors Levi and Aaron^14^. According to the Biblical narrative, Levi was the third son of the Biblical Patriarch Jacob. Levi’s given name was transformed, in time, to the Levite tribal honorific Halevi (The Levite, in Hebrew). One great-grand son of Levi, namely Aaron, was given an honorific to represent his occupation – Cohen (to serve, in Hebrew), the first high priest. Alternative theories for the origins of the Levite caste have been proposed^15^. While many contemporary Levites still use the Biblical surname form (Levi), the surname continued to evolve throughout the millennia through phonetic spelling variations (e.g. Levin, Lewicki), or through the adoption of a residency location of a specific Levite dynasty as a surname. An illustrative example of the latter is the Horowitz Rabbinical Levite dynasty^16^ established by the migration of one Levite family from Girona, Catalonia to Horovice, a small town near Prague, Czech Republic, circa 1400 CE. While claims for documented origin in medieval Spain have been made, the founder of the dynasty is considered to be Yeshayah ‘Horovsky’ Ish Horovice (1450–1514 CE)^17^. Genealogical records of the Horowitz patrilineal dynasty comprising no less than 15 subsequent generations are available^17^.

Not surprisingly, the Levite and Cohen castes have been the focus of a series of genetic studies during the past two decades using ever-expanding portions of the Y chromosome for the analysis^18–22^. First, the Cohen dynasty was studied and found to have a limited number of founding lineages that were shared between Ashkenazi and non-Ashkenazi Jews^19,21,22^. The most frequent Cohen lineage, comprising 46.1% of contemporary, self-identifying Cohen males, is found within haplogroup J1-P58, which is prevalent in the Middle East^19^. Next, it was shown that the paternal ancestry found among Ashkenazi Levites is dominated by a set of tightly evolving Y chromosome lineages falling within haplogroup R1a-M198 which was, at the time of publication, the most resolved branch known on this evolutionary path^18^. Other haplogroups reported among Ashkenazi Levites demonstrated no additional significant founding event, and the haplogroup R1a-M198 founder event was not shared with Sephardi Levites. These findings captured the attention of both scientists and laypersons, as the magnitude of the founder effect suggests that fully ~200,000 males with the tradition of Levite descent share a recent common direct male ancestor within recent historical time frames^23^. Importantly, the initial genetic analyses suggested in this first publication incorrectly attributed this Ashkenazi Levite lineage’s origin to Eastern Europe^18^. A follow up study, summarizing information from whole Y chromosome sequencing, focused specifically on this Ashkenazi Levite lineage and confirmed that that 65% of the 97 randomly assembled Ashkenazi Levites carried haplogroup R1a-M198^20^. Strikingly, the better resolved whole Y chromosome based phylogeny of haplogroup R1a, showed that 100% of these samples could be reassigned to the refined haplogroup R1a-M582. This distinctive R1a-M582 lineage was found, other than in Ashkenazi Jews, among 15.7% males self-affiliating as non-Ashkenazi Levites and, importantly, at low frequencies only in the Middle East, consistent with this location as its ancestral origin^20^.

**Figure 1:**
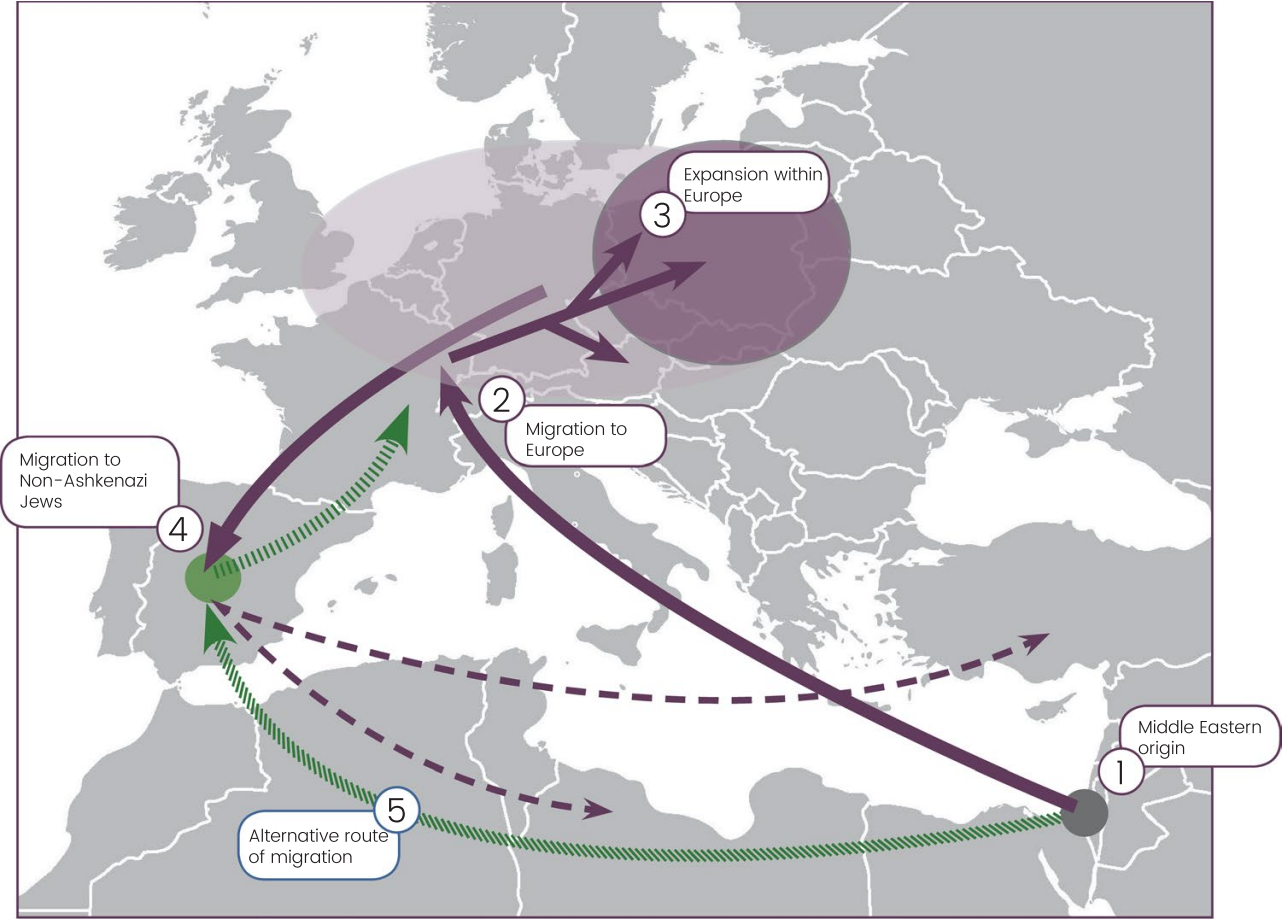
Origin and expansion of the Ashkenazi Levite Y chromosome clade. The suggested gradual movement and expansion pattern of the Ashkenazi Levite haplogroup R1a-Y2619 are denoted by ascending numerical labels. An ancestral origin in the Middle East (1) is followed by a migration route (purple arrows) paralleling the dispersal of Ashkenazi Jews to Europe (2). Expansion within the Ashkenazi Jewish population in Europe (3) is followed by a paternal gene flow of R1a-Y2619 Y chromosomes to non-Ashkenazi Jewish communities (4). A second theoretical expansion route of Levites from the Middle East to Europe (5) via the expansion of non-Ashkenazi Jews is shown but not supported by the obtained results. Map data is from ©2017 Google Maps.

While the phylogenetic origin of the R1a-M582 lineage was clarified^20^, the aim of this study is to further explore several questions that remained open regarding this founder lineage among Ashkenazi Levites. First, the limited number of whole Y chromosome sequences from Ashkenazi Levites had precluded a definitive description of their phylogenetic branch, its coalescence time and its route of entrance to Europe (Fig. 1). Second, the ancestral ties between Ashkenazi Jews self-affiliating as Levites and Ashkenazi Jews self-affiliating as non-Levites within haplogroup R1a-M582 remained elusive. Third, the existence of haplogroup R1a-M582 in both Ashkenazi and non-Ashkenazi Levites was not explained. Fourth, lack of whole Y chromosome sequences from other Ashkenazi haplogroups did not allow a comparison between a specific founding event for Ashkenazi Levites and a general expansion of Ashkenazi Jews that also affected the Ashkenazi Levites. Fifth, a comparison between the coalescence ages of the dominant Cohen priesthood J1-P58 lineage shared between Ashkenazi and non-Ashkenazi Jews and the Ashkenazi Levite lineage at the level of the whole Y chromosome sequences was yet to be conducted. Having these objectives in mind, we assembled 486 whole Y chromosome sequences from Ashkenazi Jews with a tradition of Levite descent (Supplemental Table S1), including members of the Horowitz rabbinical dynasty, Ashkenazi Jews without a tradition of Levite descent, non-Ashkenazi Jews and non-Jews. Of these, 179 are novel, including 65 R1a-M582 samples that were collected following expert genealogical input. This set of 65 samples consists of males with 56 different surnames, who claim to have an Ashkenazi Levite paternal origin. Samples were chosen to include the widest possible range of haplogroup R1a-M582 internal variation based on their previously available short tandem repeat (STR) haplotypes (Supplemental Table S2). Additional samples were included to provide the appropriate phylogenetic framework for the studied haplogroups.

## Results

### The Ashkenazi Levite phylogenetic branch

**Figure 2:**
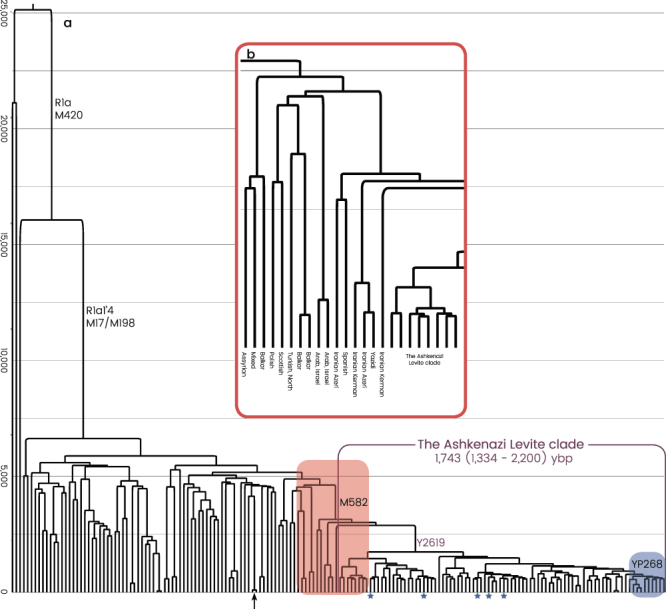
The Ashkenazi Levite clade. **(a)** Haplogroup R1a phylogeny comprising 170 samples is illustrated to nest the refined Ashkenazi Levite clade R1a-Y2619. The phylogeny and coalescence times (Y axis) were calculated using the software package BEAST v.1.7.5. The Ashkenazi Levite R1a-Y2619 clade coalesces 1,743 (1,334–2,200) ybp. Each terminal branch represents one sample (Supplemental Figure S1). The single arrow points to a sample sequenced by both the Complete Genomics and Illumina platforms. Samples highlighted by a blue star are from non-Ashkenazi Levites. The area shaded in blue represents the sub-Ashkenazi Levite clade nesting all the samples self-affiliating as belonging to the Horowitz pedigree (Fig. 3). The area shaded in red is magnified in **(b)** and details the ancestries of the individuals making the Ashkenazi paraphyletic clades within haplogroup R1a-M582.

All R1a-M582 Y chromosomes sampled from Ashkenazi Levites, non-Ashkenazi Levites, Ashkenazi non-Levites, and non-Jews with known or suspected Ashkenazi origin established a well-defined phylogenetic branch nested within haplogroup R1a-M582 and demonstrated a star-like expansion pattern (Fig. 2 and Supplemental Figure S1). The root of this branch is defined by a total of six polymorphic sites and designated according to one of the positions, R1a-Y2619 (g.6733896A>G) coalescing 1,743 (1,334–2,200) ybp (Table 1). The five non-Ashkenazi Levites and the single Iraqi Jew did not establish a distinct phylogenetic cluster but scattered within the Ashkenazi Levite samples. The sister clades of R1a-Y2619 within R1a-M582, coalescing ~3,143 (2,620–3,682) ybp, were sampled in Iranian Azeris, a Kerman, a Yazidi and one sample from Iberia. Further, the phylogeny demonstrates a rich diversity of R1a samples distributed throughout the Middle East, Anatolia, Caucasus and the Indian sub-continent, whereas East European branches represent an early split within R1a.

**Table 1:** Coalescence times of most relevant haplogroups to the Ashkenazi expansion. Bayesian estimations of ages are expressed in ybp with 95% HPD intervals.

Haplogroup	Marker	Age (Height Median)	Lower (95% Highest Posterior Density)	Upper (95% Highest Posterior Density)	Posterior
**R1a**	M420	16060	13821	18490	1.00
R1a1'4	M17/198	6633	6153	7243	1.00
R1a1'2	Z645	5884	5882	5886	1.00
R1a1	Z283	5592	5254	5836	1.00
R1a2	Z93	5474	5068	5803	1.00
R1a3	CTS4385	5019	4210	6012	1.00
R1a-M582	M582	3143	2620	3682	1.00
R1a-Y2619	Y2619	1743	1334	2200	1.00
R1a-YP268	YP268	691	555	852	0.99
**J1**	M267	18862	17243	20451	1.00
J1b	P58	10230	8922	11594	1.00
J1b-B236	B236	4874	4194	5541	0.98
J1b-B877	B877	2570	2051	3121	1.00
J1a	Z1828	6661	5719	7717	1.00
J2	M172	30968	29636	32086	1.00
J2b	M12	16750	15144	18452	1.00
J2b-M241	M241	5442	4575	6328	1.00
J2b-B239	B239	1455	843	2164	1.00
J2b-M205	M205	5454	4606	6353	1.00
J2a	M410	19081	17375	20854	1.00
J2a-M318	M318	1645	1018	2353	1.00
J2a-M92	M92	1437	928	2046	1.00
**E2**	M35	25589	23760	27506	1.00
E2a	M78	14516	13086	16004	1.00
E2a-B933	B933	1208	809	1607	1.00
E2b-Z838	Z838	1604	1147	2214	1.00
E2b-M34	M34	15715	14207	17330	1.00
E2b-PF6746	PF6746	8589	7542	9717	1.00
E2b-M123	M123	18436	16804	20117	1.00
E2b	CTS8288	20535	18885	22210	1.00
E2b-PF3780	PF3780	1476	1008	2077	1.00
E2b-B923	B923	1302	832	1943	1.00
E2c	L355	15208	13304	17164	1.00
E2c-M81	M81	2644	1752	3595	1.00
E2b’c	Z827	25258	23421	27143	0.95
E1a’b	M2	16297	14366	18245	1.00
E4	M33	22409	20091	24780	1.00
**G2**	P287_eq	22740	21494	24194	1.00
G2a	P15	19405	19403	19407	1.00
G2b	M377_eq	5757	4495	7078	1.00
G2b2	BY764	1223	786	1733	1.00
**Q**	M242	32431	31104	34008	0.95
Q3	M378	4007	3167	5007	1.00
Q3-B853	B853	1672	1163	2240	1.00
**T1a**	M70	19242	17221	21294	1.00
T1a1	L162_eq	9951	8537	11477	1.00
**L1b**	M349	8634	7205	10117	1.00
**R1b1′14**	L754	18530	17399	19575	1.00
R1b13	M73	2708	1738	3868	1.00
R1b1′12	M269	6444	5663	7245	1.00
R1b11	Z20105_eq	5677	4961	6438	1.00
R1b11-B308	B308	5378	4750	6054	0.97
R1b1′17	M412	6246	5525	7027	0.94
R1b1′9	L11_eq	5589	5011	6205	1.00
R1b1′9-B20_eq	B20_eq	4340	3550	5103	1.00
R1b1	L408	393	119	760	1.00

**Figure 3:**
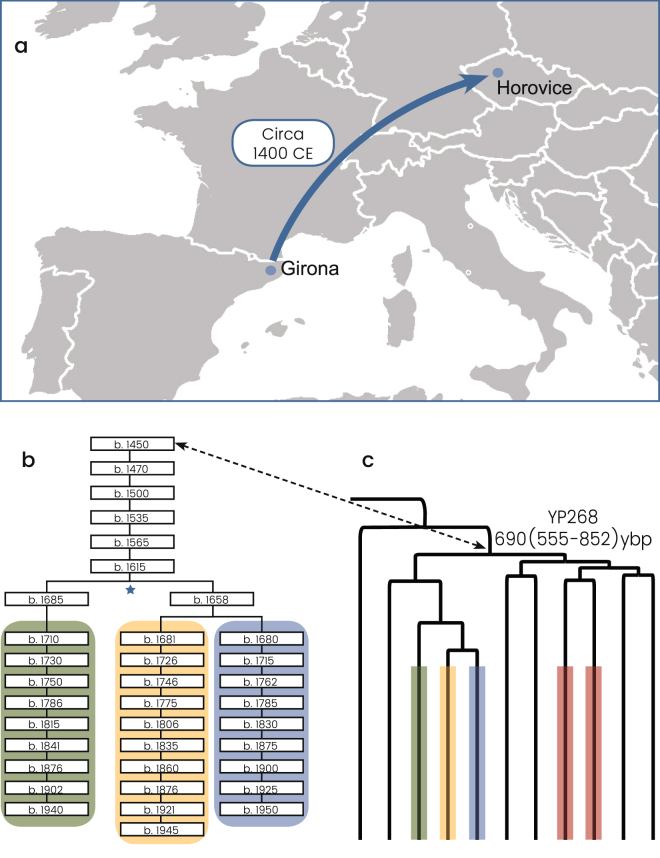
The Horowitz Levite pedigree. **(a)** The presumed migration route of the first named founder of the Horowitz pedigree from Girona to Horovice, circa 1400 CE, is shown. **(b)** A total of five individuals self-affiliated as descendants of the pedigree. Of these, three individuals supplied detailed written genealogies showing the ancestral relatedness among them. These three individuals are highlighted by the green, yellow and blue colors, and the noted birth years of their ancestors are noted. The node noted by the blue star symbol was used as an internal calibration point for the R1a phylogeny (Fig. 1). (**c**) The obtained YP268 clade phylogeny including all five Horowitz Y chromosomes is shown to coalesce 690 (555–852) ybp. The respective allocations of the three individuals comprising the written genealogies are noted by the same colors. The digits to the left of the branches denote the number of mutational events observed in each branch (Supplemental File 1). The two additional individuals are noted in red. The dashed double-headed arrow points to the YP268 node and the first named ancestor of Horowitz pedigree. Map data is from ©2017 Google Maps.

We then explored the allocation of the Horowitz Levite samples included in the phylogeny. First, the six tested Horowitz Levites were grouped into the Y chromosome haplogroup R1a-Y2619, allowing a unique glimpse into medieval Europe (Fig. 3a). The genealogic records for three of the individuals with the Horowitz surname converged to a common male ancestor born at 1615 CE or 402 ybp (Fig. 3b). The observed sequence variation between these three samples is consistent with this proposed genealogy (Fig. 3c), and accordingly, their genealogical claim could not be refuted. This prompted us to use this node as an internal calibration point. The two additional individuals affiliating with the Horowitz dynasty formed the closest paraphyletic clade R1a-YP268 to the described three sample cluster, coalescing with them 691 (555–852) ybp (Fig. 3c). The sixth sample carrying the Horowitz surname but claiming no ancestral relations with the Horowitz dynasty did not cluster with these five samples (Supplemental Figure S1).

### Additional Ashkenazi Y chromosome haplogroups

We further studied the major Y chromosome haplogroups found among Ashkenazi Jews^24^ to shed light on their origin and expansion times in comparison to the findings obtained for the R1a lineages among the Levites.

Haplogroup J grants the largest overall contribution to the Ashkenazi paternal gene pool, accounting for 38% of the total variation^19,21,22^. The haplogroup J phylogeny, including its two major sub-branches J1 and J2, is presented in Supplemental Figure S2. We first focused on J1-M267 and particularly on the Cohen lineage nested with haplogroup J1-P58^19^. A total of five Ashkenazi and five non-Ashkenazi novel Cohen J1-P58 samples were included in the analysis. Remarkably, the five Ashkenazi Cohen samples formed the tight cluster J1b-B877, shared only with one Yemenite, one Bulgarian and one Moroccan Cohen coalescing ~2,570 ybp (Table 1). Additional J1-P58 samples from Jews clustered within this branch or within other J1-P58 sub-branches. Both the paraphyletic haplogroups and the clades within which the Ashkenazi Cohen samples were nested, were of clear Middle Eastern origin. Another minor Cohen haplogroup has been previously described within haplogroup J2-M12^19^. The three J2-M12 samples included in this study share the deep clade J2b-M241 with one Albanian sample coalescing ~5,442 ybp (Table 1). Two of the three Cohen J2-M12 samples coalesced ~1,836 ybp (Supplemental Figure S2). Interestingly, the third sample shares the clade J2b-B239 with two Cochin Jews, coalescing ~1,455 ybp (Table 1). This finding provides the first evidence of an instance of shared paternal ancestry between Ashkenazi and Cochin Jews claiming to have resided in Kerala, India, since biblical times. Last, a minor, previously described Cohen J-M318 haplotype specific to the Tunisian island of Djerba^24^, is now phylogenetically fully characterized and the two included samples coalesce ~1,645 ybp (Table 1) with one mainland Tunisian Jew, whose Cohen status is unknown.

The phylogeny of haplogroup E, comprising 20.4% of the paternal variation found among Ashkenazi Jews^24^, demonstrates a complex pattern of a few, well defined, evolving sub-haplogroups (Supplemental Figure S3). As expected, the Ashkenazi samples were distributed among various sub-E haplogroups^24^. The emerging pattern is of a complex demographic history. A few well-defined recent lineages coalesce ~1,604, ~1,476, ~1,302 and ~1,208 ybp within haplogroups E-Z838, E- PF3780, E- B923 and E-B933 (Table 1), respectively, alongside deep-rooted branches suggesting their long existence within Ashkenazi Jews. Non-Ashkenazi Jewish samples found within these haplogroups primarily coalesce with the Ashkenazi samples in time periods that antedate the arrival of the latter to Northeast Europe.

Haplogroup G-M377, found in 9.7% of contemporary Ashkenazi males^24^, coalesces ~5,757 ybp (Supplemental Figure S4). The Ashkenazi samples clustered only with other Ashkenazi samples reported elsewhere and could be refined here to haplogroup G-BY764 coalescing ~1,223 ybp (Table 1). Interestingly, the closest Y chromosome within G-M377 is from a Punjabi male. The phylogeny obtained for haplogroup Q-M378 comprising 5.2% of the Ashkenazi paternal variation^24^, shows a similar pattern to that observed for haplogroup G-M377 (Supplemental Figure S5). Herein five new Jewish sequences from three Ashkenazi, one Moroccan and one Yemenite Jew are presented. The Yemenite Jewish sample seems to fall within the central Asian and Indian sub-continent variety. The three Ashkenazi samples form a tight cluster Q3-B853, coalescing ~1,672 ybp, that was shared only with a previously reported Ashkenazi sample (Table 1). The sequence from a Moroccan Jew and a previously reported sample of unclarified ancestry form the closest branch coalescing with the Ashkenazi samples ~4,007 ybp. The deep split of this Jewish cluster from its closest sister clade is ~32,431 ybp, leaving the phylogenetic origin of this lineage enigmatic. This pattern might suggest an occasional survival of remote splits within a population residing in, or transiting through, the Levant that have survived in small frequencies among contemporary Ashkenazi Jews.

The phylogeny of haplogroup T-M70 bears a few deep-rooted branches shared by Ashkenazi, non-Ashkenazi, and non-Jewish samples (Supplemental Figure S6). The diversity of this haplogroup attests to its long presence within Jewish populations at time frames that predate the Jewish Diaspora^25^.

The phylogeny of haplogroup R1b-M269 shows the presence of this haplogroup in various Jewish communities. The Ashkenazi samples clustered primarily with European R1b samples or created recently forming clusters. This pattern might be compatible with repeated introgression of non-Jewish European R1b Y chromosomes into the Ashkenazi Jewish population (Supplemental Figure S7).

## Discussion

Cumulatively, the extensive number of samples assembled herein, combined with the availability of very highly resolved paternal phylogenies extending back many generations, have enabled certain inferences about the Ashkenazi Levite haplogroup R1a founding lineage to be formulated with a high degree of confidence. A total of 71 individuals declaring an Ashkenazi Levite or Ashkenazi non-Levite paternal heritage were ascertained at the inception of the study to belong to haplogroup R1a-M582, and based on their STR profiles, to represent a broad range of the variation found within that haplogroup. Strikingly, all 71 Y chromosomes could be reassigned to a single expanding clade nested within R1a-M582 and labeled herein R1a-Y2619, following one of the six variants found at that level (Fig. 2 and Supplemental Figure S1). The only previously reported samples who shared this clade were five samples of Ashkenazi Jewish heritage for which the Levite status was not reported^26^. Accordingly, all R1a-Y2619 individuals, whether self-affiliating as Jews or non-Jews, whether Ashkenazi or non-Ashkenazi, whether Levites or non-Levites, are the direct male descendants of the paternal line of one common male ancestor who lived ~1,743 ybp (Table 1). As contemporary males from all branches of R1a-Y2619 sampled so far carry one of the many Levites surnames, it can be strongly argued that this male ancestor self-affiliated as a Levite and may have carried the patronymic surname Levite. The magnitude of the founding event can be estimated by calculating the number of contemporary individuals expected to carry an R1a-Y2619 Y chromosome. Two independent sample sets of Ashkenazi Jews in which the Levite status was unknown have similarly estimated the percentage of the R1a-Y2619 paternal haplogroup, R1a-M17/M198, in the Ashkenazi population at 9.6%^20^ and 11.5%, respectively^27^. The former paper reported that haplogroup R1a-M582 accounted for 7.9% of the total Ashkenazi population^20^. Here, we show that all Ashkenazi samples belonging to haplogroup R1a-M582 can be reclassified as R1a-Y2619. Based upon an Ashkenazi population size of ~4,000,000^13^ males, of whom about 7.9% are R1a-Y2619, there would be ~300,000 Ashkenazi males descending on their direct male line from a single relatively recent ancestor, with many of those men self-affiliating as Levite.

Two factors enabled us to achieve more accurate coalescence ages and confidence intervals than previously calculated for the Ashkenazi Levite R1a lineage^18,20^. First, the large number of whole Y chromosomes available (Supplemental Table S1), and second, the data available from the Horowitz pedigree that allowed an internal calibration point (Fig. 3). The well-documented genealogical records of the Horowitz pedigree demonstrate how robust whole Y chromosome sequences can be in reconstructing paternal ancestries. The calibration point was based on three Horowitz samples with well-documented genealogies coalescing 402 ybp. Two additional individuals, self-affiliating with the Horowitz dynasty, but without clear genealogies clustered tightly with these three samples. None of the samples in the immediate sister clades self-identified as part of the Horowitz dynasty. Strikingly, the molecular age of the node comprising all five Horowitz samples was calculated to be ~690 ybp, while the genealogical coalescence time suggests it to be 546 ybp. This suggests that the Horowitz node R1a-YP268 (g.23133909G > A) might represent the actual reconstructed whole-Y sequence of the named founder of this pedigree (Fig. 3b). However, it is important to note that the estimated coalescence ages are approximate, as a few methodological variations may lead to somewhat different results. For example, the application of our coalescence methodology to the data obtained from the Illumina platform alone, would have yielded a coalescence age of ~1,398 ybp for the Ashkenazi Levite R1a-Y2619 clade and ~645 years ybp for the R1a-YP268 clade. Such differences might be attributable to the ability to use longer overlapping stretches of the Y chromosome when comparing samples sequenced by the same machinery and parameters. We did not calculate a coalescence time based on STR variations, as we have previously shown they provide no further information for recently coalescing clades^6^.

The proposed Middle Eastern origin of the Ashkenazi Levite lineage based on what was previously a relatively limited number of reported samples^20^, can now be considered firmly validated. While the highest frequencies of haplogroup R1a are found in Eastern Europe^18,28^, our data revealed a rich variation of haplogroup R1a outside of Europe which is phylogenetically separate from the typically European R1a branches. Evidently, R1a-Y2619 is well nested within a plethora of phylogenetically close Middle Eastern sister-clades, sampled in Iranian Azeris, a Kerman, a Yazidi and one man from Iberia. This provides the needed evidence for its origin (Fig. 2 and Supplemental Figure S1). However, the exact migration pathway of R1a-Y2619 to Europe remains elusive. Most historical records suggest two major routes of Jewish migration to Europe (Fig. 1)^29,30^. Ashkenazi Jewry is considered to have been founded as the result of Jewish migration via Italy to the Rhine Valley, and then Poland. Sephardic (Spanish) Jews are considered to have migrated along with the gradual Islamic expansion to North Africa and then Spain^14^. Because R1a-Y2619 is of Middle Eastern origin, it is possible that its introduction to Europe was by either, or both, of these routes. Naturally, the strong founding event for R1a-Y2619 among Ashkenazi Jews, coupled with the presence of all known branches of R1a-Y2619 in Ashkenazi Jews, tempts to infer that its migration route from the Levant was directly related to the Ashkenazi founders. However, these facts might merely reflect an expansion within Ashkenazi Jews rather than a proof of first arrival of the R1a-Y2619 with or into the Ashkenazi population. More confusing is the fact that genealogical records of the Horowitz rabbinical dynasty, now shown to carry the R1a-Y2619 Y chromosome, suggest their presence in the Iberian Peninsula in the 15^th^ century and probably earlier (Fig. 3)^17^. In fact, repeated Jewish migrations that might have carried R1a-Y2619 Y chromosomes to Catalonia are documented since the 4th century and during the Muslim expansion to Iberia^31^. Additionally, because Catalonia was again Christian territory since 800 CE, proto-Horowitz R1a-Y2619 ancestors could also represent migration of Ashkenazi Jews to Iberia. Accordingly, the presence of R1a-Y2619 in Spain in the 15^th^ century could not establish proof for the first arrival of the R1a-Y2619 lineage to the Iberian Peninsula, as this could simply reflect repeated and unrecorded movements of Jews back and forth between Eastern and Western Europe and the Iberian Peninsula. Previous evidences from mtDNA and autosomal markers have already suggested a likely gene flow between Ashkenazi and Sephardic Jews within Europe^32–34^. Taken together, our results tend to favor a single route of entry to Europe as part of the Ashkenazi migration and expansion in Europe. Because the coalescence time of all contemporary R1a-Y2619 Levites is ~1,743 ybp, well within the time of the Roman exile Diaspora, and each of the branches of R1a-Y2619 is found in Ashkenazi Jews, our results are inconsistent with a scenario of rapid expansion in the Levant followed by a spread via multiple routes to Europe. The results from the non-Ashkenazi R1a-Y2619 Levite samples also suggest single expansion route. Had the R1a-Y2619 Levite lineage already been as widely prevalent in the Levant, the non-Ashkenazi Levites would have been expected to split from the Ashkenazi Levite samples prior to the time of the Diaspora and to form a different cluster. However, the results show that the non-Ashkenazi Levites fall within multiple relatively recently coalescing sub-branches of R1a-Y2619 (Fig. 2 and Supplemental Figure S1). This pattern is more compatible with continuous gene flow from the Ashkenazi population to the non-Ashkenazi population during the Diaspora, rather than multiple routes of entrance for haplogroup R1a-Y2619 to Europe from the Levant.

Further support for a single migration route and subsequent expansion within Ashkenazi Jews, emanates from the observed patterns of expansion of additional Ashkenazi haplogroups. Accordingly, it is important to distinguish between an Ashkenazi Levite specific founding event for haplogroup R1a-Y2619 which could have been the result of a favorable socio-economic or other status, and a general expansion of Ashkenazi founding Y chromosome lineages, including R1a-Y2619 Ashkenazi Levites. For this purpose, we have studied a handful of other haplogroups previously described to be prevalent among Ashkenazi Jews^19,24,35^. Our results show that the expansion of R1a-Y2619 among Ashkenazi Jews is not specific or unique to this haplogroup. For example, the coalescence of haplogroup G-M377 (Supplemental Figure S4) and Q-M242 (Supplemental Figure S5), known to each represent 5% of the Ashkenazi paternal variation^24^, coalesce at ~1,223 ybp and ~1,672 ybp, respectively (Table 1). The Y chromosome strategy adopted herein allowed us to resolve haplogroup E lineages into its minute sub-branches (Supplemental Figure S3). The coalescence ages of haplogroup E-Z838, E- PF3780, E- B923 and E-B933, known to cumulatively represent 20% of the Ashkenazi paternal variation^24^, were estimated at ~1,200–1,600 ybp. This pattern of multiple founding events, not observed among Spanish Jews^36^ provides further support that the R1a-Y2619 Ashkenazi Levite ancestor entered Europe via the Ashkenazi route rather than via the Jewish expansion to the Iberian Peninsula. Other patterns are also clearly visible in the Ashkenazi Jewish paternal ancestry. Haplogroup T-M70, prevalent in the Middle East, is also present among Ashkenazi Jews (Supplemental Figure S6). The genotyped samples showed deeply rooted splits probably pointing to the preservation of an ancient diversity of this haplogroup in the Levant, dating back to Pleistocene. Meanwhile, the pattern observed for haplogroup R1b-M269 (Supplemental Figure S7), prevalent in Western Europe, primarily suggests repeated punctuated introgression of European Y chromosomes to the Ashkenazi community, and is compatible with previous reports^36^.

We further compared the most frequent founding lineage found among Ashkenazi Cohen males, nested within haplogroup J1a-P58, to the Ashkenazi Levite R1a-Y2619 lineage. Evidently, members of the R1a-Y2619 Levite caste and the J1a-P58 Cohen caste do not share a common male ancestor within the time frame of the Biblical narrative. As in the case of the Ashkenazi Levites, the Ashkenazi Cohen J1a-P58 lineage formed a tight cluster nested within a Middle Eastern set of samples confirming its origin, and was shared in our study only with non-Ashkenazi Cohens (Supplemental Figure S2). However, differently from the pattern obtained for R1a-Y2619 Ashkenazi Levites, the cluster coalesces ~2,570 ybp, thus pointing to the start of its expansion in the pre-Diaspora period. Extensive full Y chromosome sequences from larger number of Cohen samples from more Jewish communities on the background of other Levantine populations, including ascertainment of family and clan specific variants would be very informative in addressing the finding that Ashkenazi and non-Ashkenazi Cohen individuals share an overlapping distribution of lineages. In particular, the study of the most dominant Cohen lineage nested with the prevalent haplogroup J1-P58, along with expert historical input, might grant critical insight to the understanding of Hebrews in the Old World. Furthermore, ancient DNA studies of the Levant may offer direct information. Indeed, a recent study revealed the presence of both J1a-P58 and J2-M12 Y-chromosomes, frequent among contemporary Jews, in two Canaanite samples date to 3,700 ybp^37^.

It is difficult to simulate the maximal number of males that would have needed to be present at the foundation of Ashkenazi Jewry to yield the strong pattern of founding events currently observed in the Ashkenazi Y chromosome pool. Fundamental indices such as frequencies of the respective haplogroups in the ancestral deme populations and the extent of introgression that has occurred throughout the generations are lacking. However, the contemporary frequencies of these Y chromosome founding lineages, in view of their very much lower frequencies in non-Jewish Middle Eastern and European populations, suggest that such lineages must have been present at the inception of Ashkenazi Jewry, when scant number of males comprised the founding population, and allowing for drift to play a role in establishment of their current high frequencies. Indeed, reconstruction of the recent Ashkenazi Jewish history from whole genomes suggested a bottleneck of merely 350 individuals^26,38^. It is important to note that while this bottleneck does not necessarily coincide with the founding effective male population size and events for Ashkenazi Jews, it does tell us that the Ashkenazi Levite R1a-Y2619 ancestor was likely among the founding males upon whom the bottleneck applied. Correspondingly, data from complete mitochondrial DNA sequences support the same notion of a limited number of major founding maternal lineages^33^.

Taken together, the magnitude of the data presented herein facilitates a near complete resolution of the genomic tale of Levites within Ashkenazi Jews. It can be strongly argued that contemporary R1a-Y2619 Ashkenazi Levites descend from a single Levite ancestor who arrived in Europe from the Levant. The expansion of his direct male lineage began in a timeframe compatible with the expansion pattern observed for several additional founding fathers of Ashkenazi Jewry. Thus, in addition to providing insight onto a single male genealogy, these findings are an important and highly resolved example of the more general demographic history of Ashkenazi male and female Jewish lineages, in which a relatively small number of founders make disproportionately large contributions to contemporary Ashkenazi population genomic variation. It can be further argued that the fact that the non-Ashkenazi Levite R1a-Y2619 men are well distributed within the Ashkenazi Levite phylogeny, rather than clustered separately, lends more credence to the scenario that the R1a-Y2619 male entered Europe with the Ashkenazi founders. The most enigmatic question – the timing and location whereby the founder of the Ashkenazi Levite R1a-Y2619 pedigree obtained Levite status – remains unresolved. This question might be beyond the scope of genetic studies using contemporary genetic variation, given the absence to date of any tested men whose lines branched off between the time of the shared direct male ancestor of all R1a-M582 men, ~3,143 ybp, and the shared direct male ancestor of the R1a-Y2619 Ashkenazi Levite men ~1,743 ybp. Future historical or archeological insights might provide the means to further investigate this issue.

## Material and Methods

### Sampling

A total of 486 samples from unrelated individuals were assembled, of which 179 are novel and 307 were previously reported (Supplemental Table S1). The study was approved by the Research Ethics Committee of the University of Tartu, Estonia and the Rambam Medical Center in Haifa, Israel. All donors provided informed consent and all experiments were performed in accordance with the relevant guidelines and regulations of the collaborating institutions. First, following expert genealogical advice, a total of 85 novel R1a samples were selected by inspecting a database of over 40,000 R1a samples available at Gene by Gene (Family Tree DNA). The samples were selected based on previously available clade diagnostic variants and STR profiles (Supplemental Table S2) aiming to genotype a broad range of R1a-M582 samples or phylogenetic adjacent sister clades^18,20^. This tier comprised 60 males with documented paternal Ashkenazi Levite ancestry, five non-Jews with known Ashkenazi Levite paternal origin, four Ashkenazi non-Levites, two non-Jews with known paternal Ashkenazi origin, one Iraqi Jew, and 13 non-Jews from the Middle East, Caucasus and Europe (Supplemental Table S1). Next, 85 additional R1a samples, of which 16 are reported for the first time in this study, were included to provide the relevant framework for the R1a phylogeny. Importantly, five of the 16 novel samples were from non-Ashkenazi Levites. Finally, a total of 316 samples, of which 78 are first reported herein, carrying haplogroups known to be prevalent among Ashkenazi Jews were selected including E-M123, E-M78, E-M81, E-M35*, G-M377, J-M12, J-M267, J-M318, J-M410, J-P58, T-M70, Q-M378, and R-M269. These samples represent the variation found among Jews against the background of a wide set of West Eurasian samples. Where available, the paternal ancestry information of these samples also notes the self-reported Jewish caste with which the donor affiliates, namely, Cohen, Levite or Israelite.

Five samples (Supplemental Table S1) self-affiliated with the Horowitz dynasty of which three presented well documented genealogies. A sixth sample carried the Horowitz family name but claimed no relation to the dynasty.

Five samples (Supplemental Table S1) were run on both the Complete Genomics and Illumina platform to reassure that the data obtained from both platforms could be assembled^6^.

### Whole Y chromosome sequencing

All novel samples were genotyped using the Illumina HiSeq. 2,500 platform following Y chromosome capture using a proprietary capture protocol available at Gene by Gene (Family Tree DNA) using the commercially available “BigY” service (https://www.FamilyTreeDNA.com/documents/bigy_targets.txt), a targeted enrichment design utilizing 67,000 capture probes for sequencing at least 10 Mbp on the Illumina HiSeq platform at >60x coverage. The targeted regions lie within the non-recombining male-specific parts of the Y chromosome.

### Mapping and calling the Y chromosome variants

For mapping and calling the raw paired-end read data we followed the best practices recommended by the SAMtools developers (http://www.htslib.org/workflow), starting from BWA MEM mapping to the GRCh37 human reference sequence, ‘decoy’ version (hs37d5), including duplicate read removal with picard-tools-2.0.1 (http://broadinstitute.github.io/picard), indel realignment with GATK-3.5 and finishing with multisample base calling by SAMtools and BCFtools^39–41^. All parameters are detailed in the Supplemental Table S3. Quality filtering was done using vcftools-0.1.12, the default filter settings were used except for the following values: base coverage >4x and <500; base quality >20; distance between SNPs >5 bp.

The novel Y chromosome capture data was combined with high coverage Y chromosomes extracted from previously published human whole genomes sequenced^6,26,42,43^ at Complete Genomics (Mountain View, California), the 1000 Genomes^44^ Consortium and the Personal Genomes Project (http://www.personalgenomes.org/).

### Filtering

We extracted the overlap between the published sequences and the newly generated data and applied our previously published custom filtering scheme^6^ that concentrates on parts of Y chromosome reachable with NGS (the ‘re-mapping filter’) and minimizes the platform bias when merging datasets. After the filtering, we ended up with 6 million bp of Y chromosome data. We also excluded insertions, deletions and multistate SNPs from the analyses, as well as SNP sites with over 5% of no-calls.

### Phylogeny reconstruction

We constructed phylogenies for each Y chromosome haplogroup. Maximum likelihood trees were constructed with RaXML^45^. All identified variants were annotated based on these trees using in-house scripts followed by manual curation. We estimated the coalescence times with the software package BEAST v.1.7.5^42^. We used a Bayesian skyline coalescent tree prior, the general time reversible (GTR) substitution model with gamma distributed rates, and a stringent clock with uniform distribution for all haplogroups was utilized. Runs were performed with a piecewise-constant coalescent model with the number of groups depending on the number of samples on the particular phylogeny, following the best practices in BEAST usage where the number of groups used is the number of samples divided by a value between 5 to 20, but having no more than 20 groups. The MCMC runs had 200 million iterations that were sampled every 3,000 steps. We ran four parallel runs with different seeds for phylogenies with more than 50 samples and two parallels for the smaller phylogenies. We visualized each BEAST run in Tracer v1.5 and confirmed that all effective sample size (ESS) values were above 200. We used previously published ages for relevant nodes^6^ as calibration points in each tree. The trees were all visualized using FigTree 1.4.2. (http://tree.bio.ed.ac.uk/software/figtree/). The BEAST-generated phylogenies present the samples comprising them, the coalescence ages of the nodal positions, and the labels of major branches or branches relevant for this paper. Annotations of the major haplogroups of interest appearing in the BEAST phylogenies follow the published nomenclature^6^ and are detailed in Supplemental File 1.

### Data Availability

The 179 whole Y chromosome sequences reported in this paper are deposited in the European Nucleotide Archive (http://www.ebi.ac.uk/ena) under the accession number PRJEB21310.

## Electronic supplementary material

Supplemental Figures S1-S7

Supplemental File 1

Supplemental Table 1

Supplemental Table 2

Supplemental Table 3

## References

[1] Hey, D. *family Names and Family History*. (Hambledon & London, 2006).

[2] Jobling MA, Tyler-Smith C (2003). The human Y chromosome: an evolutionary marker comes of age. Nature reviews. Genetics.

[3] Underhill PA, Kivisild T (2007). Use of y chromosome and mitochondrial DNA population structure in tracing human migrations. Annual review of genetics.

[4] Jobling MA, Tyler-Smith C (2017). Human Y-chromosome variation in the genome-sequencing era. Nature reviews. Genetics.

[5] Gymrek M, McGuire AL, Golan D, Halperin E, Erlich Y (2013). Identifying personal genomes by surname inference. Science.

[6] Karmin M (2015). A recent bottleneck of Y chromosome diversity coincides with a global change in culture. Genome research.

[7] Mendez FL (2013). An African American paternal lineage adds an extremely ancient root to the human Y chromosome phylogenetic tree. American journal of human genetics.

[8] Poznik GD (2016). Punctuated bursts in human male demography inferred from 1,244 worldwide Y-chromosome sequences. Nature genetics.

[9] Helgason A (2015). The Y-chromosome point mutation rate in humans. Nature genetics.

[10] Ilumae AM (2016). Human Y Chromosome Haplogroup N: A Non-trivial Time-Resolved Phylogeography that Cuts across Language Families. American journal of human genetics.

[11] Kaganoff, B. C. *A Dictionary of Jewish Names and Their History*. (Rowman and Littlefield, 2005).

[12] Guggenheimer, H. W. G., E. H. *Jewish Family Names and Their Origins: An Etymological Dictionary*. (KTAV Publishing House, 1992).

[13] Della Pergola, S. in American Jewish Year Book 2013 Vol. 113 *American Jewish Year Book* (eds Dashefsky, A. & Sheskin, I.) Ch. 6, 279–358 (Springer International Publishing, 2014).

[14] *Encyclopedia Judaica*.

[15] Leuchter, M. *The Levites and the Boundaries of Israelite Identity*. (Oxford University Press, 2017).

[16] Horowitz, T. *Toldot Mishpahat Horowit*z. (1928).

[17] The Horowitz Families Association, http://www.horowitzassociation.org/.

[18] Behar DM (2003). Multiple origins of Ashkenazi Levites: Y chromosome evidence for both Near Eastern and European ancestries. American journal of human genetics.

[19] Hammer MF (2009). Extended Y chromosome haplotypes resolve multiple and unique lineages of the Jewish priesthood. Human genetics.

[20] Rootsi S (2013). Phylogenetic applications of whole Y-chromosome sequences and the Near Eastern origin of Ashkenazi Levites. Nature communications.

[21] Skorecki K (1997). Y chromosomes of Jewish priests. Nature.

[22] Thomas MG (1998). Origins of Old Testament priests. Nature.

[23] Wexler, J. D. *Levite DNA*, LeviteDNA.org (2013).

[24] Behar DM (2004). Contrasting patterns of Y chromosome variation in Ashkenazi Jewish and host non-Jewish European populations. Human genetics.

[25] Mendez FL (2011). Increased resolution of Y chromosome haplogroup T defines relationships among populations of the Near East, Europe, and Africa. Human biology.

[26] Carmi S (2014). Sequencing an Ashkenazi reference panel supports population-targeted personal genomics and illuminates Jewish and European origins. Nature communications.

[27] Nebel A, Filon D, Faerman M, Soodyall H, Oppenheim A (2005). Y chromosome evidence for a founder effect in Ashkenazi Jews. European journal of human genetics: EJHG.

[28] Underhill PA (2015). Thephylogenetic and geographic structure of Y-chromosome haplogroup R1a. European journal of human genetics: EJHG.

[29] Ben-Sasson, H. H. *A History of the Jewish People*. (Harvard University Press, 1976).

[30] De Lange, N. *Atlas of the Jewish World*. (Phaidon Press, 1984).

[31] Forcano, M. *Història de la Catalunya Jueva: Vida i mort de les comunitats jueves de la Catalunya medieval*. (AMBIT SERVEIS EDITORIALS, S.A., 2009).

[32] Atzmon G (2010). Abraham’s children in the genome era: major Jewish diaspora populations comprise distinct genetic clusters with shared Middle Eastern Ancestry. American journal of human genetics.

[33] Behar DM (2006). The matrilineal ancestry of Ashkenazi Jewry: portrait of a recent founder event. American journal of human genetics.

[34] Behar DM (2010). The genome-wide structure of the Jewish people. Nature.

[35] Hammer MF (2000). Jewish and Middle Eastern non-Jewish populations share a common pool of Y-chromosome biallelic haplotypes. Proceedings of the National Academy of Sciences of the United States of America.

[36] Adams SM (2008). The genetic legacy of religious diversity and intolerance: paternal lineages of Christians, Jews, and Muslims in the Iberian Peninsula. American journal of human genetics.

[37] Haber M (2017). Continuity and Admixture in the Last Five Millennia of Levantine History from Ancient Canaanite and Present-Day Lebanese Genome Sequences. American journal of human genetics.

[38] Xue J, Lencz T, Darvasi A, Pe’er I, Carmi S (2017). The time and place of European admixture in Ashkenazi Jewish history. PLoS genetics.

[39] Danecek P (2011). The variant call format and VCFtools. Bioinformatics.

[40] Li H (2009). The Sequence Alignment/Map format and SAMtools. Bioinformatics.

[41] McKenna A (2010). The Genome Analysis Toolkit: a MapReduce framework for analyzing next-generation DNA sequencing data. Genome research.

[42] Drmanac R (2010). Human genome sequencing using unchained base reads on self-assembling DNA nanoarrays. Science.

[43] Lachance J (2012). Evolutionary history and adaptation from high-coverage whole-genome sequences of diverse African hunter-gatherers. Cell.

[44] Genomes Project C (2015). A global reference for human genetic variation. Nature.

[45] Stamatakis A (2014). RAxML version 8: a tool for phylogenetic analysis and post-analysis of large phylogenies. Bioinformatics.

