# Supplementary material for: The genetic variation in the R1a clade among the Ashkenazi Levites’ Y chromosome
Source: Sci Rep. 2017 Nov 2;7:14969. doi: 10.1038/s41598-017-14761-7 (PMC5668307; doi:10.1038/s41598-017-14761-7)
Supplement: Supplementary file 1 — Supplemental Figures S1-S7 [file 41598_2017_14761_MOESM1_ESM.pdf]

The genetic variation in the R1a clade among the Ashkenazi Levites' Y chromosome

Doron M Behar*[1,2], Lauri Saag[1], Monika Karmin[1], Meir G. Gover[3], Jeffrey D. Wexler[4], Luisa Fernanda Sanchez[2], Elliott Greenspan[2], Alena Kushniarevich[1,5], Oleg Davydenko[5], Hovhannes Sahakyan[1,6], Levon Yepiskoposyan[6], Alessio Boattini[7], Stefania Sarno[7], Luca Pagani[1,8], Shai Carmi[9], Shay Tzur[9,10], Ene Metspalu[1,11], Concetta Bormans[2], Karl Skorecki[10,12], Mait Metspalu[1], Siiri Rootsi[1], Richard Villems[1,11]

[1]Estonian Biocentre, Tartu 51010, Estonia

[2]Genomic Research Center, Gene by Gene, Houston 77008, Texas, USA

[3]Independent Genetic Genealogy Researcher, Savyon 5690500, Israel

[4]levitedna.org, Los Angeles 90045, California, USA

[5]Institute of Genetics and Cytology, National Academy of Sciences of Belarus, 220072, Minsk, Belarus

[6]Laboratory of Ethnogenomics, Institute of Molecular Biology of National Academy of Sciences, Yerevan, 0014, Armenia

[7]Department of Biological, Geological and Environmental Sciences, University of Bologna, Bologna, 40126, Italy

[8]APE Lab, Dept. of Biology, University of Padova, 35121, Padova, Italy

[9]Braun School of Public Health and Community Medicine, The Hebrew University of Jerusalem, Jerusalem, 9112102, Israel

[10]Rambam Health Care Campus, Haifa 3109601, Israel

[11]Department of Evolutionary Biology, Institute of Molecular and Cell Biology University of Tartu, Tartu 51010, Estonia

[12]Ruth and Bruce Rappaport Faculty of Medicine, Technion-Israel Institute of Technology, Haifa, 3109601, Israel

*Correspondence and requests for materials should be addressed to D.M.B. (email: behardm@genebygene.com)

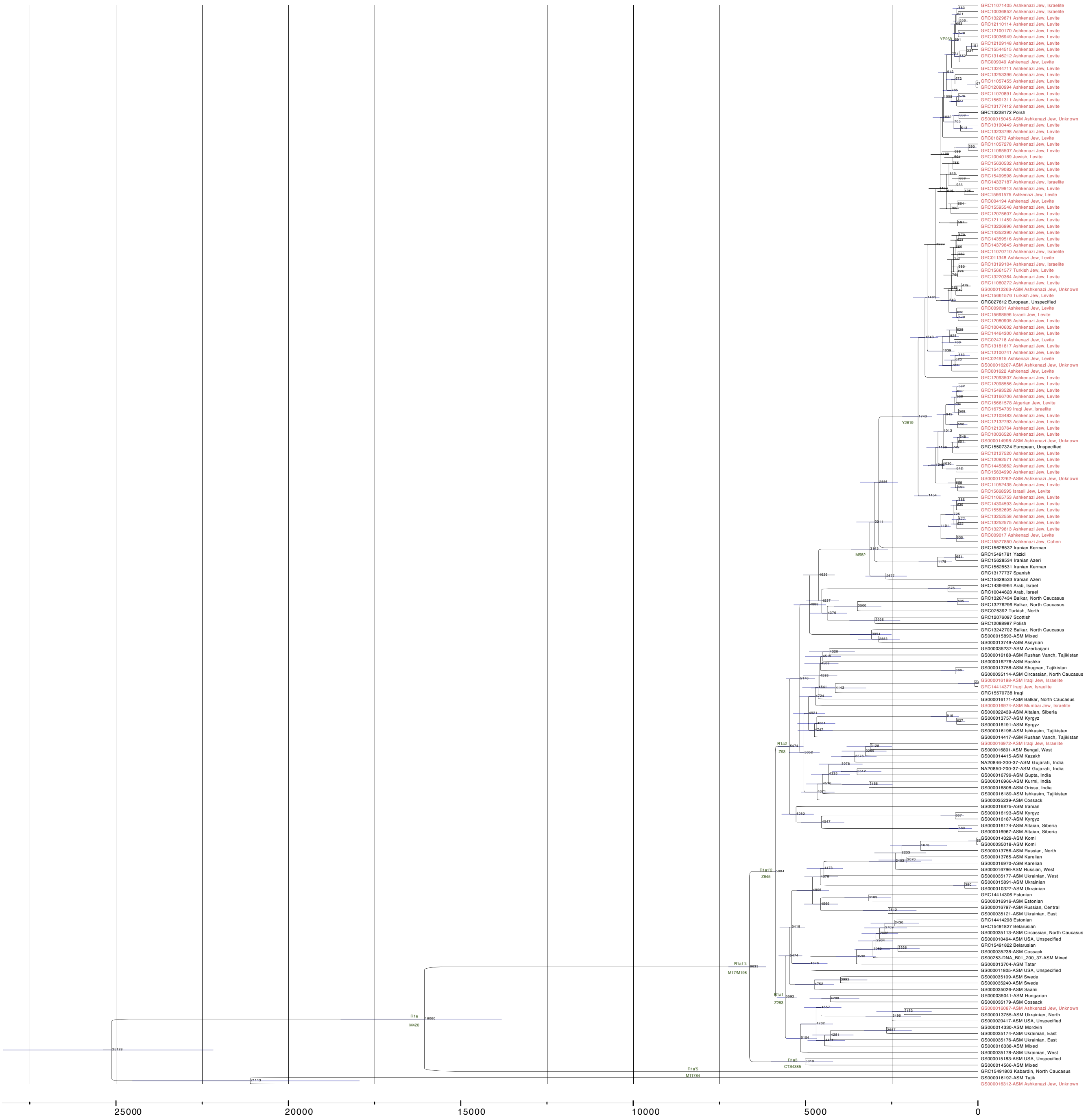

Figure S1. Haplogroup R1a phylogeny as reconstructed by BEAST with estimated coalescence ages of the nodes. Haplogroup annotations and marker names are noted along branches of interest. Coalescence ages are marked on the bifurcating nodes with bars representing the 95% confidence intervals. Individuals with Jewish ancestry are marked in red.

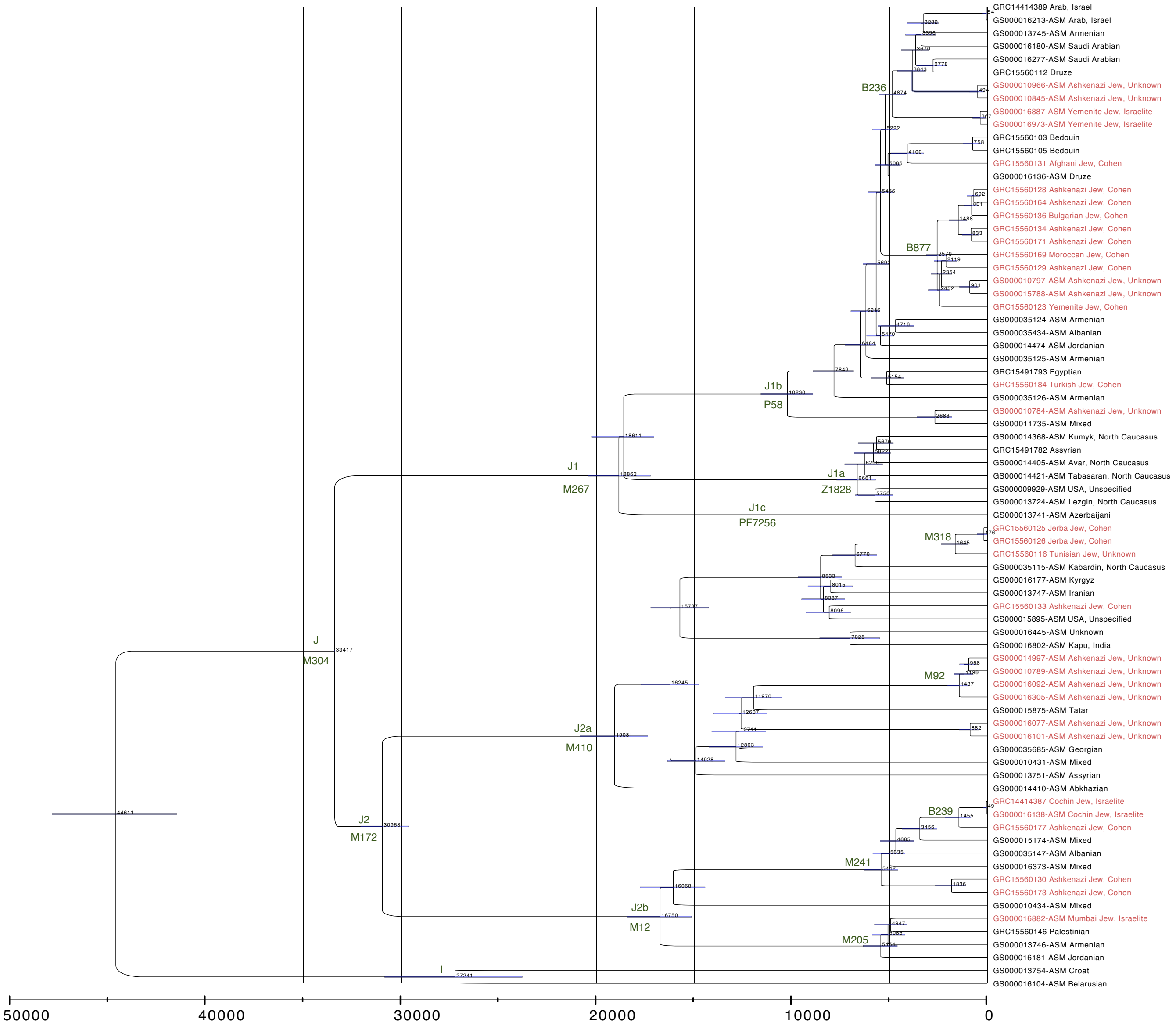

Figure S2. Haplogroup J phylogeny as reconstructed by BEAST with estimated coalescence ages of the nodes. Haplogroup annotations and marker names are noted along branches of interest. Coalescence ages are marked on the bifurcating nodes with bars representing the 95% confidence intervals. Individuals with Jewish ancestry are marked in red.

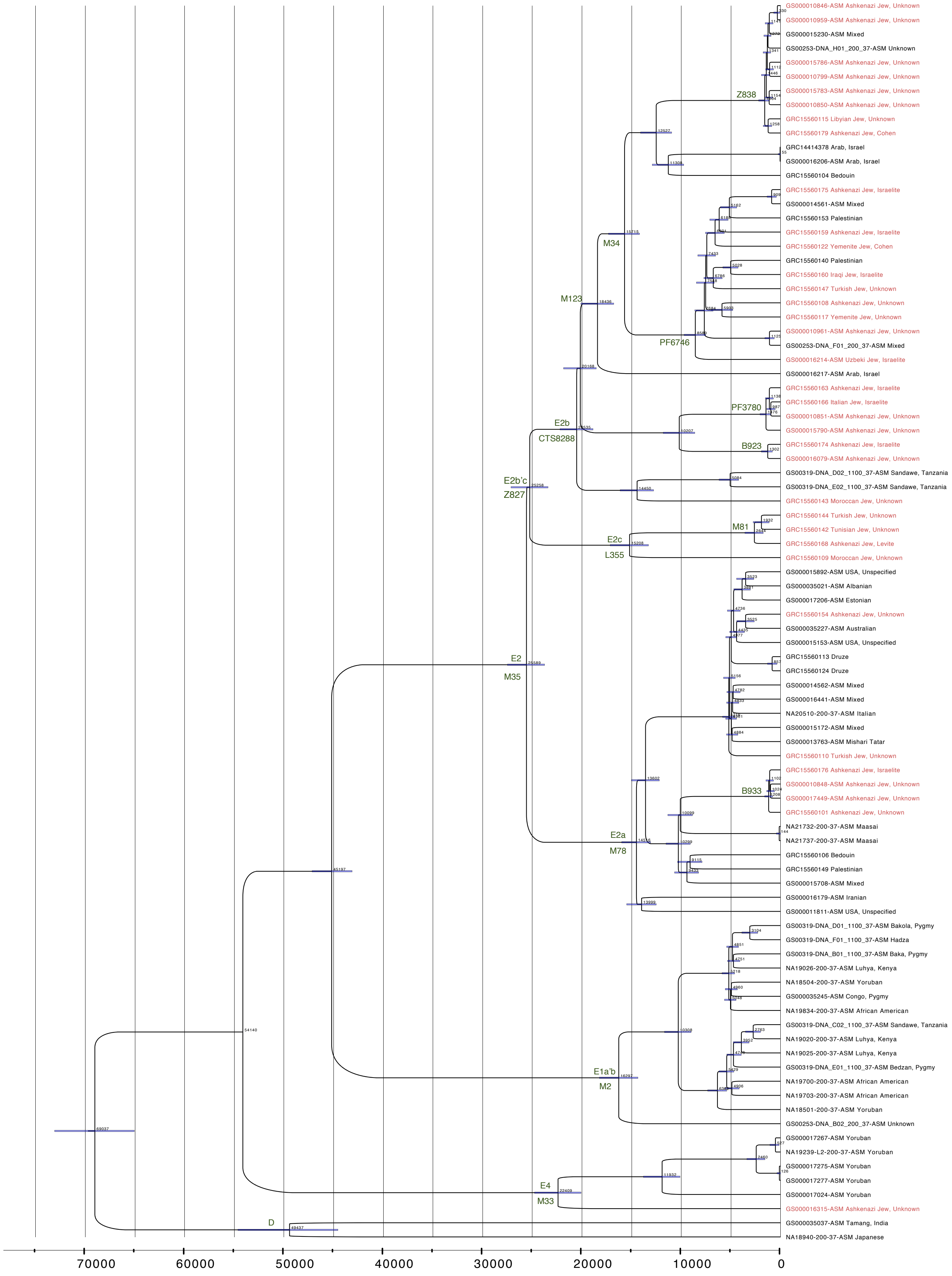

Figure S3. Haplogroup E phylogeny as reconstructed by BEAST with estimated coalescence ages of the nodes. Haplogroup annotations and marker names are noted along branches of interest. Coalescence ages are marked on the bifurcating nodes with bars representing the 95% confidence intervals. Individuals with Jewish ancestry are marked in red.

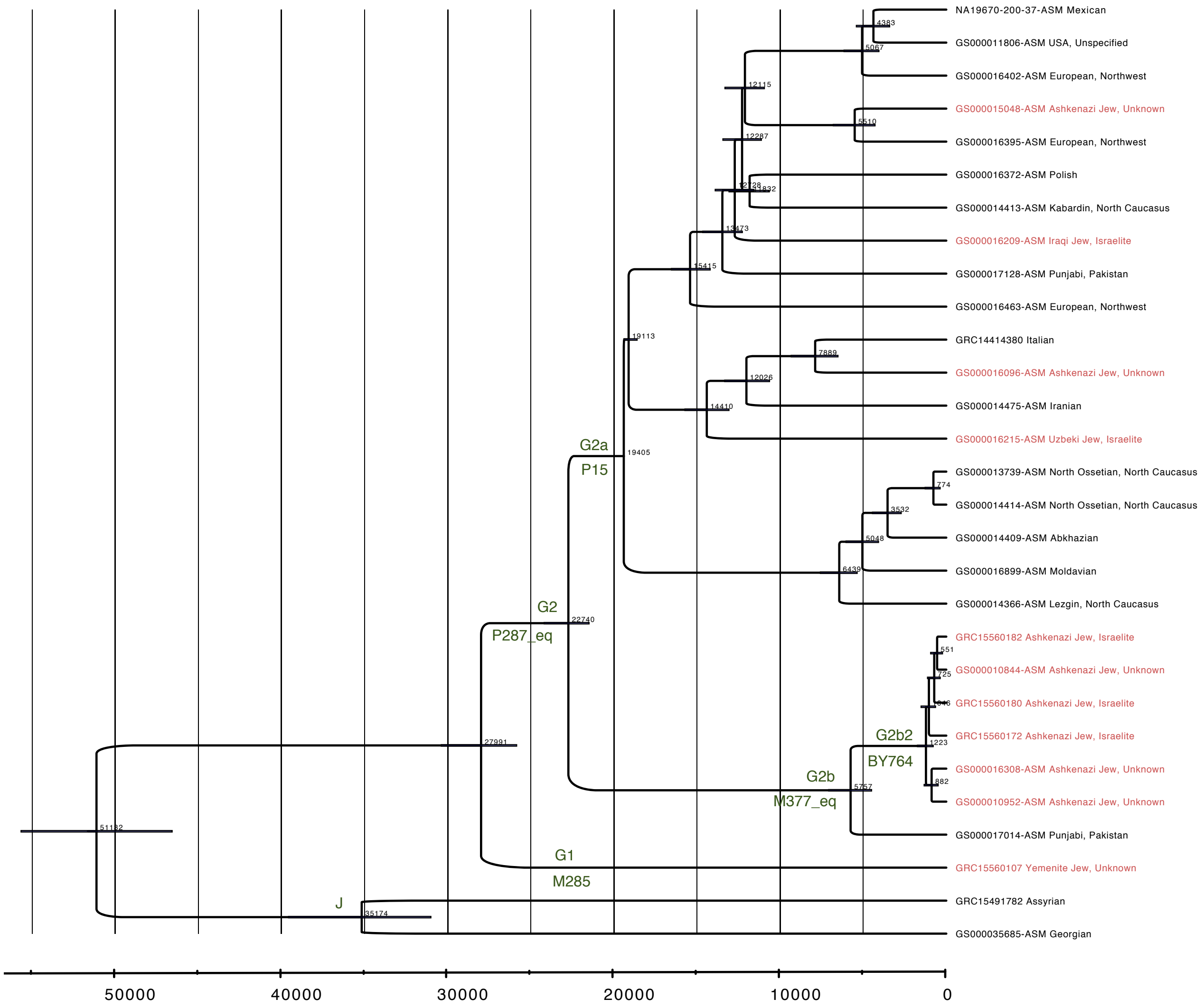

Figure S4. Haplogroup G phylogeny as reconstructed by BEAST with estimated coalescence ages of the nodes. Haplogroup annotations and marker names are noted along branches of interest. Coalescence ages are marked on the bifurcating nodes with bars representing the 95% confidence intervals. Individuals with Jewish ancestry are marked in red.

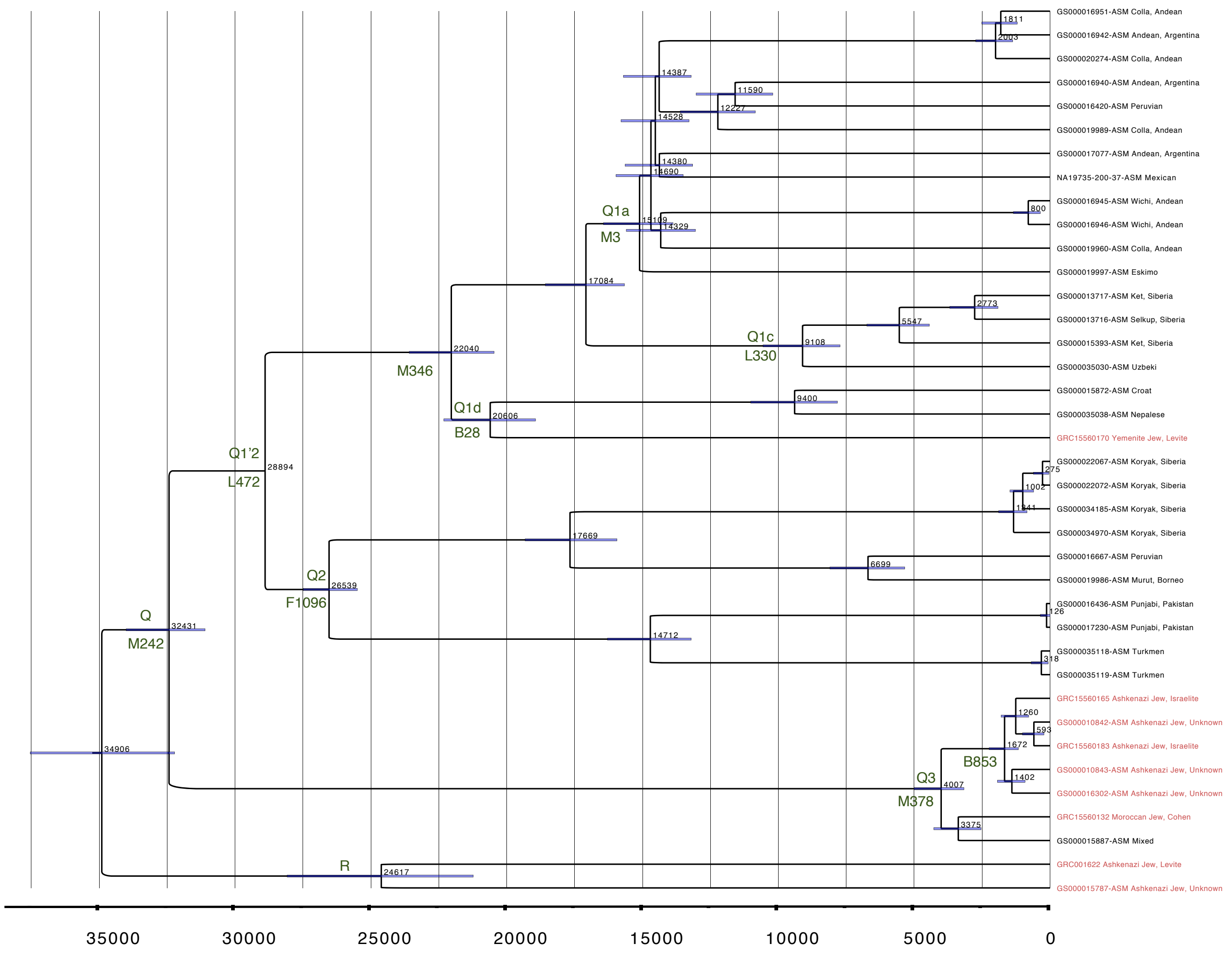

Figure S5. Haplogroup Q phylogeny as reconstructed by BEAST with estimated coalescence ages of the nodes. Haplogroup annotations and marker names are noted along branches of interest. Coalescence ages are marked on the bifurcating nodes with bars representing the 95% confidence intervals. Individuals with Jewish ancestry are marked in red.

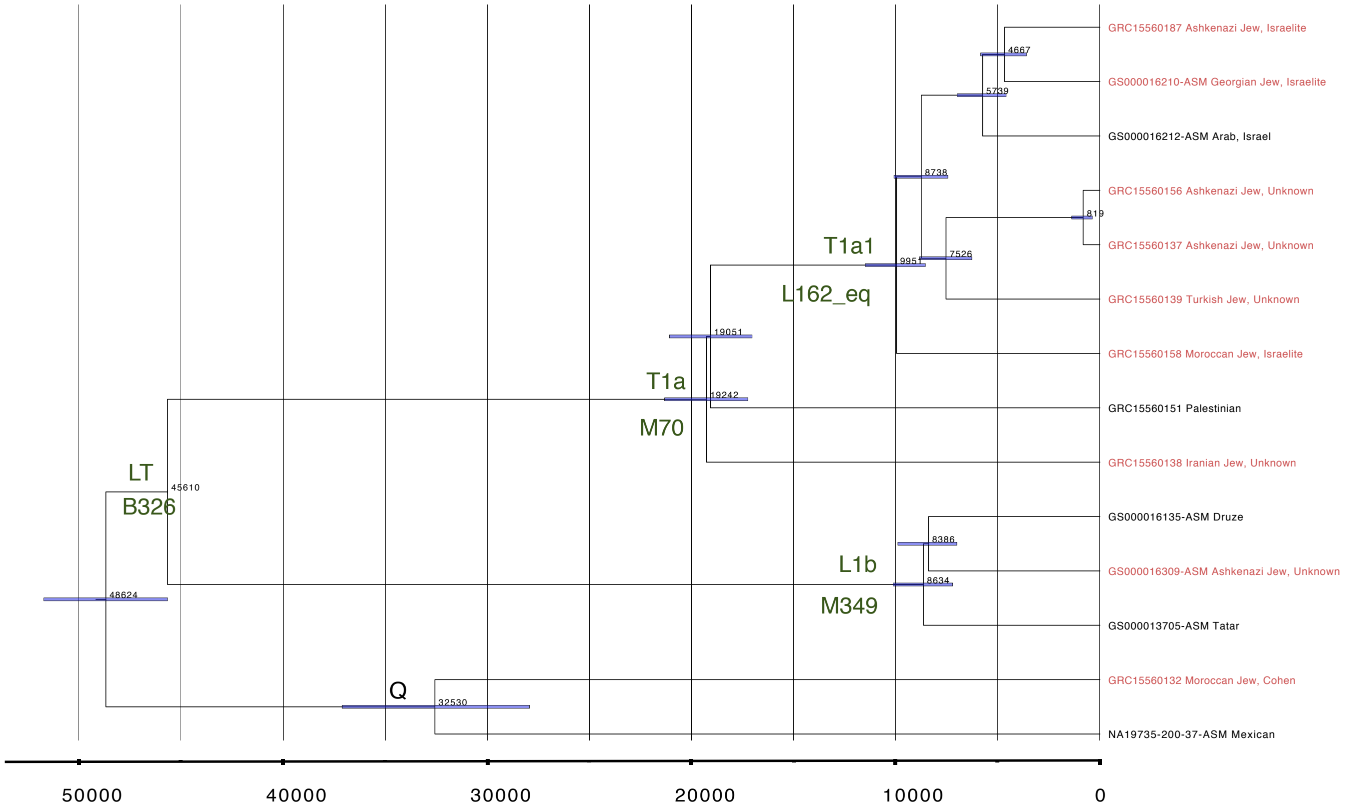

Figure S6. Haplogroup LT phylogeny as reconstructed by BEAST with estimated coalescence ages of the nodes. Haplogroup annotations and marker names are noted along branches of interest. Coalescence ages are marked on the bifurcating nodes with bars representing the 95% confidence intervals. Individuals with Jewish ancestry are marked in red.

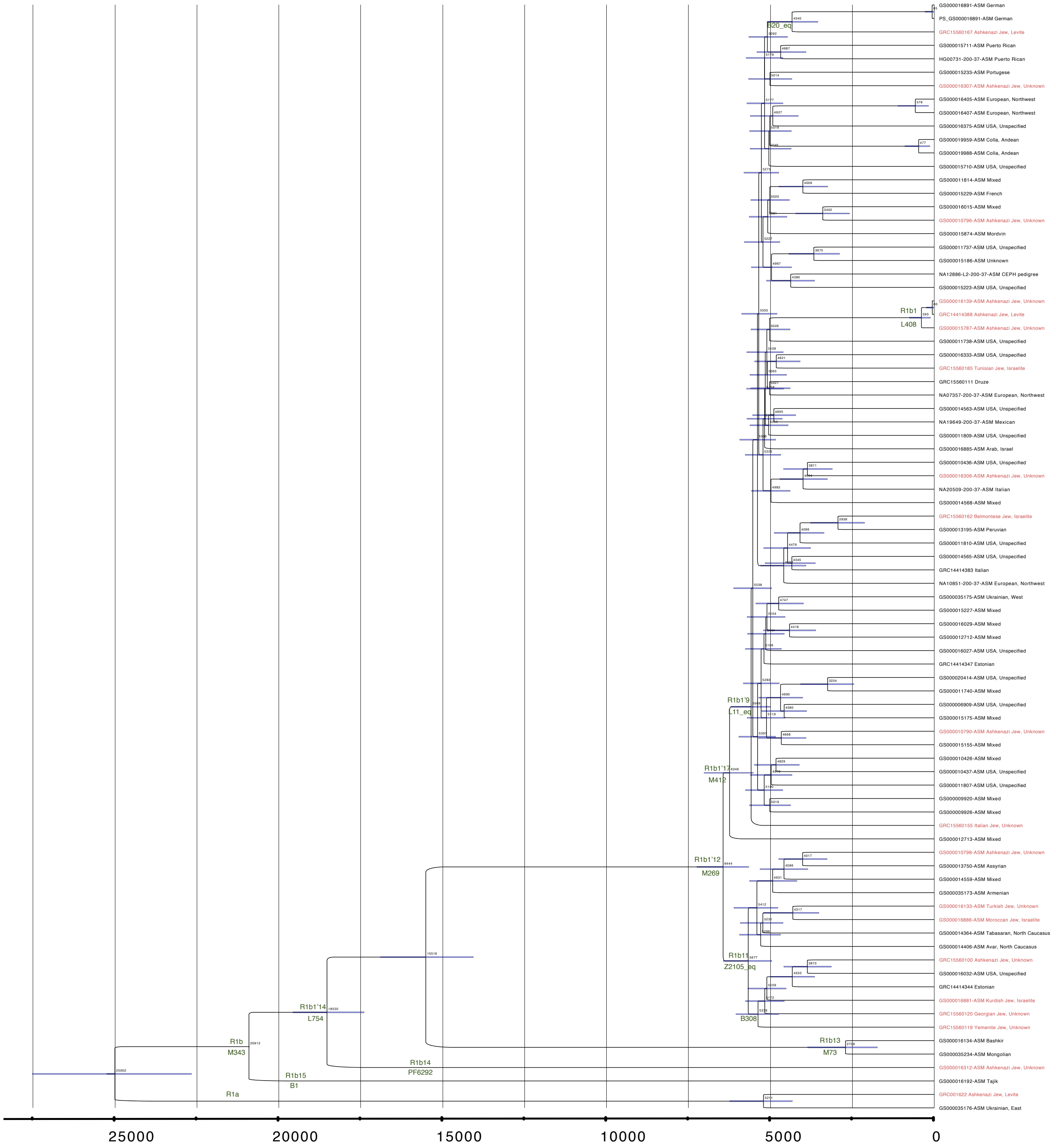

Figure S7. Haplogroup R1b phylogeny as reconstructed by BEAST with estimated coalescence ages of the nodes. Haplogroup annotations and marker names are noted along branches of interest. Coalescence ages are marked on the bifurcating nodes with bars representing the 95% confidence intervals. Individuals with Jewish ancestry are marked in red.
